# Associations of Family Meals with Adolescent Perception of Family Relationship and Compliance with Parental Guidance in Hong Kong: Results of a Representative Cross-Sectional Survey

**DOI:** 10.3390/ijerph18105402

**Published:** 2021-05-19

**Authors:** Rosa S. Wong, Keith T. S. Tung, Wilfred H. S. Wong, Frederick K. W. Ho, Winnie W. Y. Tso, Paul S. F. Yip, Carlos K. H. Wong, Susan Y. S. Fan, Patrick Ip

**Affiliations:** 1 Department of Paediatrics & Adolescent Medicine, The University of Hong Kong, HKSAR, Hong Kong, China; rosawg@connect.hku.hk (R.S.W.); keith-tung@connect.hku.hk (K.T.S.T.); whswong@hku.hk (W.H.S.W.); wytso@hku.hk (W.W.Y.T.); 2 Institute of Health and Wellbeing, University of Glasgow, Glasgow G12 8RZ, UK; Frederick.Ho@glasgow.ac.uk; 3 Department of Social Work and Social Administration, The University of Hong Kong, HKSAR, Hong Kong, China; sfpyip@hku.hk; 4 Department of Family Medicine and Primary Care, The University of Hong Kong, HKSAR, Hong Kong, China; carlosho@hku.hk; 5 Department of Pharmacology and Pharmacy, The University of Hong Kong, HKSAR, Hong Kong, China; 6 The Family Planning Association of Hong Kong, HKSAR, Hong Kong, China; sfan@famplan.org.hk

**Keywords:** adolescent development, family functioning, family meal, family dinner, family breakfast

## Abstract

Family meals are beneficial for adolescent development, but evidence from Chinese populations has been limited. This study aimed to examine the associations between family meal frequency and adolescent perception of family relationship and compliance with parental guidance in Hong Kong. During the period from October to December 2016, a stratified random sample of 3359 students were recruited from 25 secondary schools in Hong Kong. Students completed questionnaires about family characteristics, relationship quality, and meal frequency by paper-and-pencil in class. Multiple regression analyses were conducted to examine the associations between family meal frequency and perceived family relationship and compliance with parental guidance overall and by subgroups. After adjusting for sociodemographic and school confounders, family breakfast and dinner frequency were significantly associated with adolescent compliance (breakfast: B = 0.07, *p* < 0.001; dinner: B 0.07, *p* < 0.001) and perception of family relationship (breakfast: B = 0.10, *p* < 0.001; dinner: B = 0.25, *p* < 0.001). Risk factors for infrequent family meals included older age, not born in Hong Kong, less educated fathers, and unmarried parents. Our findings support the associations of regular family meals with adolescent perception of high family bond and compliance with parental guidance. Interventions are needed to enhance quality family meal interactions in disadvantaged families.

## 1. Introduction

Adolescence is known as a period of “storm and stress” [1]. It is also a period of transition from dependency on parents to independence, autonomy and maturity. Adolescents’ increased need for autonomy and non-compliant behaviors such as reluctance to follow parental rules and decisions may result in conflicts with parents [2]. Notably, the relationship between parent-adolescent conflicts and adolescent problems can be bidirectional [3] and could be stronger in collectivistic societies that emphasize interpersonal obligation. In Hong Kong, where young people are influenced by both westernized and traditional Chinese cultures, adolescents value autonomy, but they are also expected to display filial piety. The disagreement between adolescent personal preferences and parental values and expectations may not only distort family relationship but also have negative effects on adolescent psychological well-being [4]. Regular parent-adolescent communication is an important first step to reduce stress in the parent-adolescent relationship.

There has been more research and data complied over the past decade related to the role of eating family meals during childhood and adolescence. The common idea of family meal in the literature is that “various family members are together sharing a meal” [5]. Family mealtimes are valuable because they provide a good setting in which adolescents and their parents can talk about family issues and personal life events [6]. The positive outcomes that regular family meals can contribute to, such as improved adolescent wellbeing, have been found to be independent of the effect of other family factors [7]. Furthermore, research has reported that parental warmth is a strong predictor of compliant child behavior, especially when combined with appropriate levels of support and behavioral control [8]. Family mealtimes provide a good occasion for parents to show care and support to their children. However, little has been done to investigate the effect of regular family meals on adolescent compliance with parental guidance. 

Social support is a well-known buffer against psychological and social problems in adolescents [9]. While peer support has been regarded as the key support for adolescents [10], emerging evidence has highlighted the importance of support from parents for optimizing health and performance in adolescence [11]. Indeed, it has been reported that poor relationships with parents in childhood and adolescence are associated with greater odds of health and behavioral problems later in life [4,12]. On the other hand, frequent and open communication between parents and children can increase mutual understanding of each other thereby enhancing family relationship [13].

Most research on family meals have been conducted in western populations. There is limited information on the impact of family meal on adolescent perception of family relationship and compliance with parental guidance in Chinese populations. Furthermore, family meals have been studied as a general construct in previous studies. The independent and specific effects of family breakfast and dinner on adolescent perceptions and behavior have been underexplored. This study thus aimed to (1) examine the associations between family meal frequency and adolescent perception of family relationship and compliance with parental guidance in a representative Hong Kong Chinese youth sample; (2) identify the sociodemographic characteristics of those adolescents who rarely ate family dinners and breakfasts; and (3) compare the extent to which family meal frequency was related to adolescent perception of family relationship and compliance with parental guidance between population subgroups.

## 2. Methods

### 2.1. Study Design and Participants

This cross-sectional study used part of the survey data from the 2016 Youth Sexuality Study (YSS) conducted by the Family Planning Association of Hong Kong (FPYHK) between October and December in 2016. The YSS is a territory-wide serial survey conducted by the FPYHK every 5 years since 1981. It collects information about sexual knowledge, relationship issue, family relationship and behaviors among youth in Hong Kong. Researchers and policymakers can make use of the data to examine patterns and trends in various issues over time. Previous YSS results have been reported in several publications [14,15].

Students aged 12–17 years studying in grade 7 to 12 from 25 schools were recruited proportionally to their representation in the four major regions of Hong Kong (Hong Kong Island, Kowloon, New Territories West and New Territories East). Clustered random sampling, with school as the sampling unit, was used for selection of schools. Upon generating the school list, an invitation letter was sent to the selected schools. If the selected school declined our invitation, there was no replacement. On the other hand, if the selected school did not respond to our invitation, we sought their responses by making attempts to contact the school to explain our study objectives.

A total of 25 schools from the four regions in Hong Kong were approached and agreed to participate in this study. Upon obtaining the school agreement for participation, one class was randomly selected from each form of the school. Students from the selected class were surveyed using a self-administered structured questionnaire on family characteristics and other items such as their relationships with parents and other family members as well as family meal frequency. A teacher was present to administer the survey and to address students’ concerns and issues regarding the questionnaire items. All respondents were informed that the survey was conducted anonymously and confidentially to maximize the number of valid responses. Total 4073 students were recruited. After excluding students with over 10% missing and invalid responses such as providing two answers for those questions allowing only a single answer, 3359 students completed the survey, yielding a response rate of 82.5%.

### 2.2. Measures

#### 2.2.1. Sociodemographic Characteristics

Participants were asked to provide information on family characteristics such as parental educational level and marital status as well as personal information such as age, place of birth, and ethnicity.

#### 2.2.2. Frequency of Family Meals (i.e., Eating Breakfasts and/or Dinners Together as a Family) in the Past Week

The weekly frequency of eating family dinners and/or breakfasts was assessed using the following open-ended questionnaire items:

Item 1: In the past week, how often did you eat breakfasts with your family members (including your parents, grandparents, and/or siblings)?

Item 2: In the past week, how often did you eat dinners with your family members (including your parents, grandparents, and/or siblings)?

#### 2.2.3. Family Functioning

There were four questionnaire items related to family functioning. The items measure four aspects of family functioning (family life happiness, relationship with parents, compliance with parental guidance, and relationship between parents):

Item 1: Do you think your family life is happy? (family life happiness)

Item 2: How do you rate your relationship quality with parents? (relationship with parents)

Item 3: How do you rate the relationship quality between your parents? (relationship between parents)

Item 4: Do you comply with your parents’ rules and wishes? (compliance with parental guidance)

Each item is rated on a 5-point Likert scale with higher score indicating more favorable responses. The first three items can be summated to a composite score, ranging from 0 to 20, referred as perceived family relationship which reflects the extent to which the relationships between family members are positive with mutual support and respect for each other. In this study, the Cronbach’s alpha for this composite measure was 0.81.

### 2.3. Ethical Approval

This study and the consent procedures were approved by the ethical committee of the Institutional Review Board of the University of Hong Kong/Hospital Authority Hong Kong West Cluster (Ref no.: UW 17-504).

### 2.4. Data Analysis

All analyses were conducted using the Statistical Package for Social Sciences (SPSS, version 24.0, IBM Corp, Armonk, NY, USA). As missing data represented less than 5% of the whole dataset, listwise deletion was used to handle this missing data. To test our hypotheses and allow for the identification and characterization of those adolescents who were most likely to have infrequent family meals [16], the frequency of family breakfasts and family dinners was dichotomized separately into high and low by assigning the value of 1 to the bottom quarter for the low frequency group and the value of 0 to all others for the high frequency group. Descriptive statistics were then calculated to describe the sociodemographic characteristics of the total and subgroup samples. Multiple logistic regression analyses were conducted to examine the associations between sociodemographic characteristics and infrequent family breakfasts and dinners. Multiple linear regression analyses were also conducted to test whether adolescent compliance and perceived family relationship were associated with family breakfast and dinner frequency. Further subgroup analyses were performed with respects to the sociodemographic factors that showed significant associations with infrequent family breakfast and dinner. Based on prior evidence, children’s ethnicity, place of birth, current school, age and gender and parental educational level and marital status were included as covariates in all regression analyses. Statistical significance was denoted by a *p*-value of less than 0.05.

## 3. Results

### 3.1. Sample Characteristics

Total 3359 students from 25 schools agreed to participate in this study. Table 1 shows the sociodemographic characteristics of the respondents. Among the respondents, 1920 (57.2%) were boys and 1439 (42.8%) were girls. The mean age was 14.8 years (S.D. = 1.60). 1386 (41.3%) were in the young age group (12–14 years) and 1973 (58.7%) were in the older age group (15–17 years). 2579 (76.78%) had married parents at the time of survey. In terms of parental educational level, around half of the mothers and the fathers finished secondary education, respectively. For the frequency of family breakfasts, 800 (23.8%) had family breakfasts every day, 1055 (31.4%) had none, and the remaining had family breakfasts at least once in the past week. For the frequency of family dinners, 1867 (55.6%) reported eating family dinners every day, and only 108 (3.2%) had no family dinner in the past week.

Based on the family breakfast frequency distribution, the bottom quarter were respondents who had no family breakfast in the past week (*n* = 1055), referred as the infrequent family breakfast group in this study. On the other hand, the bottom quarter of the family dinner frequency distribution were respondents who had family dinners five times or less in the past week (*n* = 1050), referred as the infrequent family dinner group in this study. A majority of students in both groups were the older ones (infrequent family breakfast: 68.8%; infrequent family dinner: 68.6%). Most of the students in both groups reported their mothers and fathers to have attained secondary education or below (mothers: 66.0% in the infrequent breakfast group, 64.5% in the infrequent dinner group; fathers: 61.7% in the infrequent breakfast group, 57.3% in the infrequent dinner group). Moreover, the proportion of separated/divorced/widowed parents was higher in the infrequent family breakfast group (31.4%) and dinner group (33.4%) compared to the overall sample (23.0%).

### 3.2. Sociodemographic Correlates of Infrequent Family Breakfasts and Dinners

Table 2 shows the results of logistic regression models between sociodemographic variables and infrequent family breakfasts and dinners. After adjusting for confounders, students aged 12–14 were less likely to have infrequent family breakfasts (adjusted odd ratio, aOR = 0.58, *p* < 0.001) and dinners (aOR = 0.62, *p* < 0.001) compared to students aged 15–17. Students with married parents were less likely to be in the infrequent family breakfast (aOR = 0.56, *p* < 0.001) and dinner (aOR = 0.42, *p* < 0.001) group compared to those with non-married parents. Compared to the educated father group, students with less educated fathers were more likely to have infrequent family breakfasts (aOR = 1.76, *p* < 0.01). Maternal education level appeared to have weak relationship with infrequent family breakfast or dinner group membership. On the other hand, compared to students born outside Hong Kong, those born in Hong Kong were more likely to have infrequent family dinners (aOR = 1.31, *p* = 0.02).

### 3.3. Associations of Family Breakfast Frequency with Adolescent Perception of Family Relationship and Compliance with Parental Guidance

Table 3 shows the associations of family breakfast frequency with adolescent compliance and perceived family relationship overall and by subgroups. The results of whole group analyses showed that after adjusting for sociodemographic confounders and family dinner frequency, family breakfast frequency was significantly and positively associated with compliance with parental guidance (B = 0.07, *p* < 0.001) and perceived family relationship (B = 0.10, *p* < 0.001). Stronger associations were observed in younger students (Compliance: age 12–14 B = 0.07, *p* = 0.001 vs. age 15-17 B = 0.065, *p* < 0.001; Relationship: age 12–14 B = 0.13, *p* < 0.001 vs. age 15–17 B = 0.08, *p* = 0.01), those born outside Hong Kong (Compliance: in Hong Kong B = 0.07, *p* < 0.001 vs. outside Hong Kong B = 0.08, *p* = 0.003; Relationship: in Hong Kong B = 0.10, *p* < 0.001 vs. outside Hong Kong B = 0.12, *p* = 0.01), and those with non-married parents (Compliance: married B = 0.07, *p* < 0.001 vs. separated/divorced/widowed B = 0.09, *p* = 0.03; Relationship: married B = 0.10, *p* < 0.001 vs. separated/divorced/widowed B = 0.15, *p* = 0.04). For paternal education level, the associations were both significant only in the father attaining secondary education or above group (Compliance: B = 0.07, *p* < 0.001; Relationship: B = 0.09, *p* < 0.001).

### 3.4. Associations of Family Dinner Frequency with Adolescent Perception of Family Relationship and Compliance with Parental Guidance

Table 4 shows the associations of family dinner frequency with adolescent compliance and perceived family relationship overall and by subgroups. The results of whole group analyses showed that after adjusting for sociodemographic confounders and family breakfast frequency, family dinner frequency was significantly and positively associated with adolescent compliance (B = 0.07, *p* < 0.001) and perceived family relationship (B = 0.25, *p* < 0.001). Subgroup analyses found that the associations were significant in all groups except students of non-married parents and those born outside Hong Kong. Stronger associations with compliance were observed in younger students (age 12–14 B = 0.10, *p* < 0.001 vs. age 15–17 B = 0.06, *p* < 0.001), but the association with family relationship was comparable between age groups (age 12–14 B = 0.258, *p* < 0001 vs. age 15–17 B = 0.263, *p* < 0.001). For paternal education level, the association with compliance was stronger in those of less educated fathers (primary education or below B = 0.10, *p* = 0.04 vs. secondary education or above B = 0.06, *p* = 0.004), whereas the association with family relationship was stronger in those of more educated fathers (primary education or below B = 0.24, *p* = 0.01 vs. secondary education or above B = 0.26, *p* < 0.001).

## 4. Discussion

This study investigated the extent to which family breakfast and dinner frequency was associated with adolescent perception of family relationship and compliance with parental guidance in a representative Hong Kong Chinese youth sample. We found that the strength and significance of the associations differed by family socio-demographics. The risk factors for infrequent family breakfast were older age, separated/divorced/ widowed parents, and less educated fathers, whereas the risk factors for infrequent family dinner were older age, separated/divorced/widowed parents, and not born in Hong Kong. Consistent with previous western findings that regular mealtime interactions had positive effects on family functioning [17], our study demonstrated that the frequency of eating family breakfast and dinner was significantly associated with adolescent perception of family relationship and compliance with parental guidance. The associations were generally stronger for younger students and those with non-married parents. Notably, the associations between regular family breakfasts and dinners and compliance with parental guidance were stronger among youth of more educated fathers. Moreover, we observed that approximately half of the students were in both infrequent family breakfast and dinner groups.

This finding of a large proportion of students having infrequent family meals is alarming because evidence suggests that infrequent family meal may pose risks to children and adolescents and can worsen family functioning [7,17,18,19]. Indeed, infrequent family meals could be an indicator of limited family interactions and communication, suggesting that those adolescents who rarely eat family meals may lack adequate family care and support. Furthermore, our study found that older adolescents had a particularly high risk for infrequent family meals. This declining trend in family meal frequency over the adolescent years has been documented in previous studies [7,20]. A previous study reported that 62% of the 9–12 graders in the United States had family dinners 5 times or less per week [7]. In our study, approximately 36% of adolescents aged 15–17 years reported this frequency, suggesting that family dinners are still more common among adolescents in Chinese than western societies. In another population-based study of 4746 adolescents in the United States, the researchers observed gender differences in family meal frequency, with boys reporting more frequent family meals [20]. However, we did not observe such gender differences, possibly because eating family meals tend to be equally common for boys and girls in Chinese populations [21].

In addition to adolescent age, those factors such as paternal educational level, parental marital status, and place of birth were also found to be associated with family meal frequency. Specifically, infrequent family breakfasts were more common in adolescents of less educated fathers, whereas adolescents with separated/divorced/widowed parents were more likely to have infrequent family breakfasts and dinners compared to those with married parents. Consistent with this finding, a previous study of 37,832 individuals in the US reported that single men had lower odds of eating at all with children and having a family dinner compared to partnered/married males [22]. These differences may potentially be attributed to the irregular hours of shift work which has been commonly reported by socioeconomically disadvantaged parents [23]. Another common job feature for parents in these disadvantaged families is the long work hours which may hinder parents from eating regular family meals with their children [24]. However, in the same study, partnered/married woman had increased odds of eating at all with children compared to their partnered/married male counterparts [22]. Future research should explore reasons for such parental gender differences in their tendency to eat meals with children.

We also observed higher family breakfast and dinner frequency among the native Hong Kong-born adolescents. A possible reason for this observation is that a majority of students born outside Hong Kong were Mainland China immigrants. Numerous studies have documented that mainland immigrants generally have low incomes and limited education [25] and are not well accepted by the local-born in Hong Kong [26]. Because of these social and economic disadvantages, immigrant parents tend to work in low-wage occupations and sectors, which may in turn affect their family mealtimes with children.

Although family meal frequency has been used as a proxy variable of family connectedness [27], few studies have examined the associations of family meal frequency with adolescent perception of family relationship and compliance with parental guidance across different sociodemographic subgroups. We found that adolescents of educated fathers benefited more from regular family breakfasts and dinners. This could be because compared to less educated fathers, educated fathers might have more knowledge and resources to develop and maintain enjoyable mealtime interactions with adolescents, which would likely enhance family relationship and adolescent compliance with parental requests. The findings of no association between family dinner frequency and adolescent compliance with parental guidance in students born outside Hong Kong and those of non-married parents suggest that mealtime interactions in disadvantaged families might not be as good as those in better-off families. The quality and quantity of family meals should thus be considered in future assessment of family environment and adolescent behavior. Future research should also disentangle the mechanism underlying the positive effect of eating family meals on adolescent behavior. In addition, more studies should be done to unravel the effect of regular family breakfasts on long-term child and adolescent development.

This study has several strengths and limitations. First, this study used a representative sample of Hong Kong Chinese adolescents and hence the results are reflective of the relationship between family meal frequency and adolescent perception of family relationship and compliance with parental guidance in the Hong Kong general population. Moreover, given that half of the students were in both infrequent family breakfast and dinner groups, mutual adjustment approach was used to analyze the independent effect of family breakfast and dinner frequency. However, this study used simple questions to collect student self-reports of family meal frequency over the past week and family situation which may involve response bias. Second, this study used cross-sectional data and thus we cannot infer causal relationship between the variables of interest. Third, although we found associations between family meal frequency and adolescent perception of family relationship and compliance with parental guidance, there might be other parenting factors associated with family meals that contribute to these adolescent perceptions and behavioral outcomes. Future research is therefore needed to elucidate the effect of interplay between family meals and other parenting factors on adolescent development.

## 5. Conclusions

In line with the family and parenting literature, our findings support the associations of regular family meals with adolescent perception of high family bond and compliance with parental guidance. Furthermore, this study reveals that adolescents from disadvantaged families tend to benefit less from regular family breakfasts and dinners. Interventions are needed to enhance positive mealtime communication and interactions particularly for those at-risk families. Future research should also explore other environmental and parenting factors which might interact with family meal patterns to influence adolescent perceptions, health, and behavior.

## Figures and Tables

**Table 1:** Sample characteristics.

Sociodemographic Factors	All (*N* = 3359)	Infrequent Family Breakfast (*N* = 1055) ^a^	Infrequent Family Dinner (*N* = 1030) ^b^
	*N* (%)	*N* (%)	*N* (%)
Gender			
Male	1920 (57.16%)	596 (56.49%)	576 (55.92%)
Female	1439 (42.84%)	459 (43.51%)	454 (44.08%)
Age group			
12–14	1386 (41.26%)	329 (31.18%)	323 (31.36%)
15–17	1973 (58.74%)	726 (68.82%)	707 (68.64%)
Maternal educational level			
Primary education or below	465 (13.84%)	182 (17.25%)	148 (14.4%)
Secondary education	1676 (49.90%)	514 (48.72%)	516 (50.1%)
Tertiary education or above	457 (13.61%)	117 (11.09%)	140 (13.6%)
Missing	761 (22.65%)	242 (22.94%)	226 (21.94%)
Paternal educational level			
Primary education or below	387 (11.52%)	157 (14.88%)	128 (12.43%)
Secondary education	1585 (47.19%)	494 (46.82%)	462 (44.85%)
Tertiary education or above	564 (16.79%)	142 (13.46%)	171 (16.6%)
Missing	823 (24.50%)	262 (24.83%)	269 (26.12%)
Parental marital status			
Married	2579 (76.78%)	723 (68.53%)	684 (66.41%)
Unmarried	774 (23.04%)	331 (31.37%)	344 (33.40%)
Missing	6 (0.18%)	1 (0.09%)	2 (0.19%)
Place of birth			
Hong Kong	2404 (71.57%)	746 (70.71%)	728 (69.33%)
Outside Hong Kong	913 (27.18%)	298 (28.25%)	291 (27.71%)
Missing	42 (1.25%)	11 (1.04%)	11 (1.07%)
Frequency of having breakfasts with family members (per week)			
Never	1055 (31.41%)	1055 (100.00%)	515 (50.00%)
1 time	362 (10.78%)	-	166 (16.12%)
2 times	562 (16.73%)	-	155 (15.05%)
3 times	124 (3.69%)	-	46 (4.47%)
4 times	54 (1.61%)	-	16 (1.55%)
5 times	240 (7.14%)	-	67 (6.50%)
6 times	107 (3.19%)	-	13 (1.26%)
Everyday	800 (23.82%)	-	49 (4.76%)
Missing	55 (1.63%)	-	3 (0.29%)
Frequency of having dinners with family members (per week)			
Never	108 (3.22%)	96 (9.10%)	108 (10.49%)
1 time	120 (3.57%)	69 (6.54%)	120 (11.65%)
2 times	146 (4.35%)	73 (6.92%)	146 (14.17%)
3 times	145 (4.32%)	79 (7.49%)	145 (14.08%)
4 times	166 (4.94%)	69 (6.54%)	166 (16.12%)
5 times	345 (10.27%)	129 (12.23%)	345 (33.50%)
6 times	395 (11.76%)	137 (12.99%)	-
Everyday	1867 (55.58%)	400 (37.91%)	-
Missing	67 (1.99%)	3 (0.28%)	-

^a^ 0 time per week; ^b^ 5 times or less per week.

**Table 2:** Logistic regression analyses of the associations between sociodemographic factors and infrequent family breakfasts and dinners.

Sociodemographic Factors	Infrequent Family Breakfasts ^a^			Infrequent Family Dinners ^b^		
	aOR ^#^	95% CI	*p*	aOR ^#^	95% CI	*p*
Age group						
12–14	0.58	(0.46, 0.74)	<0.001	0.62	(0.49, 0.79)	<0.001
15–17	1.00	(reference)	-	1.00	(reference)	-
Paternal educational level						
Primary school or below	1.76	(1.24, 2.49)	<0.01	1.06	(0.74, 1.52)	0.74
Secondary school	1.29	(0.97, 1.70)	0.08	1.00	(0.76, 1.32)	0.98
Tertiary level or above	1.00	(reference)	-	1.00	(reference)	-
Maternal educational level						
Primary education or below	1.12	(0.78, 1.60)	0.56	0.87	(0.60, 1.26)	0.46
Secondary education	0.96	(0.72, 1.29)	0.80	0.95	(0.71, 1.28)	0.76
Tertiary education or above	1.00	(reference)	-	1.00	(reference)	-
Gender						
Male	1.03	(0.86, 1.25)	0.73	0.91	(0.75, 1.10)	0.32
Female	1.00	(reference)	-	1.00	(reference)	-
Place of birth						
Hong Kong	1.05	(0.84, 1.32)	0.64	1.31	(1.04, 1.65)	0.02
Non-Hong Kong	1.00	(reference)	-	1.00	(reference)	-
Parents’ marital status						
Married	0.56	(0.44, 0.70)	<0.001	0.42	(0.33, 0.53)	<0.001
Separated/Divorced/Widowed	1.00	(reference)	-	1.00	(reference)	-
Ethnicity						
Chinese	0.98	(0.53, 1.81)	0.95	0.69	(0.38, 1.24)	0.21
Non-Chinese	1.00	(reference)	-	1.00	(reference)	-

Note: ^#^ All the above factors and enrolled school were included in the same model. ^a^ 0 time per week. ^b^ 5 times or less per week.

**Table 3:** Linear regression analyses of the association between family breakfast frequency and adolescent perception of family relationship and compliance with parental guidance.

	Compliance with Parental Guidance			Perceived Family Relationship		
	B ^#^	95% CI	*p*	B ^#^	95% CI	*p*
Family breakfast frequency						
OVERALL	0.07	(0.04, 0.10)	<0.001	0.10	(0.06, 0.15)	<0.001
Subgroup analysis						
Age group						
12–14	0.07	(0.03, 0.12)	0.001	0.13	(0.06, 0.21)	<0.001
15–17	0.06	(0.03, 0.10)	<0.001	0.08	(0.02, 0.15)	0.01
Paternal education level						
Primary education or below	0.07	(−0.01, 0.14)	0.08	0.15	(0.02, 0.28)	0.02
Secondary education or above	0.07	(0.04, 0.10)	<0.001	0.09	(0.04, 0.15)	<0.001
Parental marital status						
Married	0.07	(0.04, 0.10)	<0.001	0.10	(0.05, 0.15)	<0.001
Separated/Divorced/Widowed	0.09	(0.01, 0.17)	0.03	0.15	(0.10, 0.30)	0.04
Place of birth						
Hong Kong	0.07	(0.03, 0.10)	<0.001	0.10	(0.04, 0.16)	<0.001
Outside Hong Kong	0.08	(0.03, 0.14)	0.003	0.12	(0.02, 0.21)	0.01

Notes: ^#^ Models adjusted for place of birth, enrolled school, ethnicity, age and gender, parental educational level, marital status and family dinner frequency.

**Table 4:** Linear regression analyses of the association between family dinner frequency and adolescent perception of family relationship and compliance with parental guidance.

	Compliance with Parental Guidance			Perceived Family Relationship		
	B ^#^	95% CI	*p*	B ^#^	95% CI	*p*
Family dinner frequency						
OVERALL	0.07	(0.03, 0.11)	<0.001	0.25	(0.18, 0.33)	<0.001
Subgroup analysis						
Age group						
12–14	0.10	(0.03, 0.17)	0.01	0.26	(0.14, 0.38)	<0.001
15–17	0.06	(0.01, 0.11)	0.02	0.26	(0.17, 0.35)	<0.001
Paternal education level						
Primary education or below	0.10	(0.003, 0.21)	0.04	0.24	(0.07, 0.42)	0.01
Secondary education or above	0.06	(0.02, 0.11)	0.004	0.26	(0.18, 0.34)	<0.001
Parental marital status						
Married	0.06	(0.02, 0.11)	0.01	0.25	(0.17, 0.33)	<0.001
Separated/Divorced/Widowed	0.08	(−0.01, 0.17)	0.08	0.27	(0.11, 0.43)	<0.001
Place of birth						
Hong Kong	0.08	(0.03, 0.13)	0.001	0.26	(0.17, 0.34)	<0.001
Outside Hong Kong	0.04	(−0.04, 0.12)	0.33	0.25	(0.11, 0.38)	<0.001

Notes: ^#^ Models adjusted for place of birth, enrolled school, ethnicity, age and gender, parental educational level, marital status and family breakfast frequency.

## Data Availability

The data that support the findings of this study are available on request from the corresponding author.
